# Implementation of a “real‐world” learning health system: Results from the evaluation of the Connected Health Cities programme

**DOI:** 10.1002/lrh2.10224

**Published:** 2020-02-26

**Authors:** Stephanie Steels, John Ainsworth, Tjeerd P. van Staa

**Affiliations:** ^1^ Health e‐Research Centre, School of Health Sciences, Faculty of Biology, Medicine and Health The University of Manchester Manchester UK; ^2^ Utrecht Institute for Pharmaceutical Sciences Utrecht University Utrecht The Netherlands; ^3^ Faculty of Health, Psychology and Social Care Manchester Metropolitan University Manchester UK

**Keywords:** evaluation, learning health system, LHS infrastructure, quality improvement

## Abstract

**Background:**

The “learning health system” has been proposed to deliver better outcomes for patients and communities by analyzing routinely captured health information and feeding back results to clinical staff. This approach has been piloted in the Connected Health Cities (CHC) programme in four regions in the North of England. This paper presents the results of the evaluation of this program conducted between February and December 2018.

**Methods:**

Fifty nine semistructured interviews were completed with a mix of CHC programme staff and external partners who had contributed to the delivery of the CHC programme. Interviews were audio recorded and transcribed verbatim. This also included the review of project documentation including project reports and minutes of project group meetings, in addition to a short online survey that was completed by 31 members of CHC programme staff. Data were analyzed thematically.

**Results:**

Two overarching themes emerged through the thematic analysis of participant interview: (a) challenges in the implementation of learning health system pathways, and (b) benefits to the CHC approach for both staff and patients. In particular, time constraints in delivering an ambitious program of work, data quality, and accessibility, as well as the long‐term sustainability of the CHC programme were noted as key challenges in implementing a LHS at scale.

**Conclusions:**

The findings from this evaluation provide valuable insight into creating learning health system at scale, including the potential benefits and likely challenges.

**Abbreviations**
AHSN: Academic Health Science Network
CCG: Clinical Commissioning Group
CHC: Connected Health Cities
CY: Connected Yorkshire
DHSC: Department of Health and Social Care
GDPR: General Data Protection Regulation
GM: Greater Manchester
HEI: Higher Education Institute
HRA: Health Research Authority
IG: Information Governance
I.T: Information Technology
LHS: Learning Health System
NENC: North East North Cumbria
NHS: National Health Service
NHSA: Northern Health Science Alliance
NWC: North West Coast
PPI: Patient and Public Involvement
REC: Research Ethics Committee
R&D: Research and Development
TRE: Trusted Research Environment

## 1 INTRODUCTION

The use of routinely collected health and social care data has the potential to drive forward improvements in health outcomes.^1^, ^2^, ^3^ This is especially important in the United Kingdom where an aging population increases in life expectancy and rapidly changing patterns of chronic disease have led to an increased demand in health and social care services. At the same time, the amount of health data being collected and stored is vast, while the technology and analytic tools needed to analyze “big data” has been developed.^4^, ^5^

In the North of England, there is an ever‐increasing health gap. These deep‐rooted and persistent inequalities have resulted in Northern populations regularly found to be less healthy than those who live in the South.^6^ This health gap can be found across all social groups and among both men and women.^7^ Furthermore, there is a 2‐year life expectancy gap between those who live in the North and the rest of England, with premature death rates 20% higher for those living in the North across all age groups.^6^, ^8^

*“A learning healthcare system is one that is designed to generate and apply the best evidence for the collaborative healthcare choices of each patient and provider; to drive the process of discovery as a natural outgrowth of patient care; and to ensure innovation, quality, safety, and value in health care”* ^9^ Thus, a “learning health system” should be able to deliver better outcomes for patients and communities by analyzing routinely captured health information and feeding back results to clinical staff.^10^, ^11^ This approach is being piloted in the Connected Health Cities (CHC) programme in four regions in the North of England to begin to address health inequalities across the health and care system and to reduce unjustified variations in health outcomes.

The CHC programme is a £20 million Department of Health and Social Care (DHSC) funded Northern Health Science Alliance led program. It is being delivered by a consortium of academia, NHS organizations, and industry partners across four regions in the North of England. These include Greater Manchester, North West Coast, Yorkshire, and the North East and North Cumbria. Each of the four regions was tasked with establishing a Learning Health System (LHS), using patient data to create and test innovative improvements for a variety of clinical pathways over a period of 3 years.

The CHC programme started in January 2016, with seven core deliverables set out by the DHSC forming the basis for establishing four regional LHS. These are presented in Table 1. In 2018, the final year of program delivery, an evaluation was commissioned to provide an assessment of the CHC programme in relation to progress toward these seven deliverables. However, in doing so, two key issues were raised.

**TABLE 1:** The Connected Health City programme deliverables

Deliverable	Description of deliverable
Deliverable 1	The establishment of data sharing strategies and data sharing agreements for each CHC region.
Deliverable 2	The establishment and delivery of governance arrangement for the sharing and usage of data for each CHC region, across the North and the United Kingdom.
Deliverable 3	The optimization of Ark workforce arrangements, including the identification of long‐term CPD requirements the establishment of new skill bases.
Deliverable 4	The creation of the Ark as an analytical platform for investigating linked data.
Deliverable 5	The analysis of eight care pathways, identification of any pathway variations, and proposals for any improvements if possible.
Deliverable 6	The creation and implementation of frameworks for potential integration with R&D partners and the future rising of Foreign Direct Investment.
Deliverable 7	The production of a CHC business model suitable for scaling across the North and sustainable for delivery in the NHS.

First, while the number of pathways varies for each region, the CHC programme was tasked with developing at least two pathways per region. Our funders requested that eight care pathways were included as part of the evaluation process; however, there are more than 16 in delivery. Table 2 shows the four CHC regions in relation to the eight care pathways chosen for inclusion in the evaluation with a brief overview of the work planned for each pathway.

**TABLE 2:** Description of care pathways included for evaluation, by region

Connected Health City region	Title of care pathway	Objectives of care pathway	Description of care pathway
Connected Yorkshire	Supporting community care and reducing demand on A&E services	To link de‐identified routine NHS data to describe a detailed profile of patient demand across both prehospital, primary care and hospital emergency, and urgent care settings in Yorkshire.	To collect routine NHS data from a number of emergency and urgent care (EUC) providers and link the data to provide a coherent picture of EUC demand.
	Safer prescribing for frailty	To reduce inappropriate polypharmacy for people with frailty.	To work with GPs to change behaviors related to deprescribing for older people with moderate or severe frailty as identified by electronic Frailty Index scores. This includes developing interventions using which apply evidenced tools to support deprescribing.
Greater Manchester	BRIT—Using data to tackle antibiotic resistance	To provide the NHS and clinical care teams with better information on what is happening and who is getting antibiotics. To assist in determining whether the use of antibiotics is reasonable given local resistance patterns to antibiotics	Analysis of patient records from GPs for effectiveness of antibiotic prescribing in general practices. This includes the development of a DataLab feeding back advanced analytics to clinical staff and policy makers and the evaluation of interventions to optimize prescribing.
	Using technology and data to improve the diagnosis and treatment of stroke	Improve the recognition of stroke by paramedics to maximize the proportion of acute stroke patients taken directly to a specialist stroke center for timely expert care and minimizing the number of nonstroke patients entering the stroke pathway. Provide timely and focused referral to neurosurgery for patients in Greater Manchester with stroke caused by a brain hemorrhage. Ensure that all patients get all the right treatments that they need to reduce the risk of another stroke when they are discharged from hospital.	To improve stroke recognition by paramedics by linking ambulance data to data at Salford Royal; using primary and secondary care data to create a large cohort of stroke and TIA patients for creating a predictive model of patients who are at high risk of stroke; and using acute trust data to identify predictive factors of early deterioration and death.
North East North Cumbria	Predictive modeling for unplanned care	To develop predictive modelling tools for unplanned care forecasting to support demand management and service planning in relevant health and social care services.	To produce statistical models that can be used by health/local authority/other analytics teams to produce daily forecasts up to 6 mo in advance with the pertinent associated uncertainties and variations in urgent and emergency care.
	SILVER: Smart Interventions for Local Vulnerable Families	To develop data sharing agreements to allow the linking of existing health data across multiple health agencies via one platform that provides recommendations to key workers.	To link data across multiple agencies including health (physical and mental), social care, criminal justice, housing, and education to develop a more complete Learning Health System.
North West coast	Development of a learning system for alcohol	To be able to inform health professionals about local clinical care. To define best care or treatments, implement and demonstrate benefits.	Improving the way information is collected, analyzed and shared between agencies and service users to bring opportunities for news was to respond collectively.
	Development of a learning system for unplanned care	To improve how data is used to enhance patient care admitted to hospital for emergency care.	Linking NHS data with social services data to improve the care pathway for patients with COPD and epilepsy.

Second, in designing the evaluation, it became clear that no bench marking data or any other form of monitoring data or information had been formally collected during the program delivery period. Therefore, it would be difficult to accurately measure impact and calculate any economic benefits or potential savings to the health system since the CHC programme's inception in January 2016.

Furthermore, halfway through the data collection period, the DHSC changed the CHC programme deliverables and scope of the evaluation. To enable this, the project as a whole was extended beyond the original end date of December 2018 to end of 2019. As a result, the original evaluation changed from being an “end of program” evaluation to an interim evaluation.

The purpose of this article is to present the results emerging from the interim evaluation with an emphasis on the emerging benefits and key challenges faced by the CHC programme in implementing learning health system across the North of England.

## 2 METHODS

This evaluation took place between February 2018 and December 2018. To begin with, an inception meeting with the project management group and review of key project documentation took place. Semistructured informational interviews were conducted with key CHC staff from each of the pathways and the central CHC hub in mid‐February to late April 2018. A total of 28 key staff participated in these interviews. The aim of these informational interviews was to establish which two pathways would be put forward for evaluation; what they considered to be the greatest challenges; any issues they foresaw; successes and unintentional outcomes of the CHC and a consideration of future challenges with regard to the CHC programme deliverables.

This allowed the scope of the evaluation to be determined, the evaluation questions set, and the development of an online survey and semistructured interview. Ethical review was granted by The University of Manchester Research Ethics Committee in May 2018 (Ref: 2018‐3923‐6106).

### 2.1 Data collection

In May 2018, lead pathway and regional partners were sent the online survey to be cascaded to staff working on the CHC programme. This was to gain a broad understanding of CHC staff experiences across the different pathways in relation to the program deliverables, as well as their views on the challenges, benefits, impact, and successes. A reminder was sent out 4 weeks after the original mailing, with pathway leads reminding staff to complete the e‐survey.

Qualitative research was undertaken with a selection of CHC staff for each pathway and region. In late May 2018, initial semistructured confidential interviews were conducted. Detailed discussions with a cross section of pathway and regional staff enabled the development of a balanced narrative of key achievements and challenges across the CHC programme. These were then used to inform recommendations and the development of case studies. Interviews were carried out until October 2018. In order to consider the wider benefits and challenges of the CHC programme within each region, interviews were also conducted with a number of stakeholders who sit within the CHC programme but were not directly involved in activities in the eight care pathways or regional activities.

### 2.2 Data analysis

Our data analysis utilized a thematic approach^12^, ^13^ where data from the documentary review, survey, and interview data were triangulated to ensure consistency in our findings. Thematic analysis is a widely used method to identify, organize, analyze, and report patterns or themes within qualitative research data.^14^ Themes were compared across participants and documents with data analysis taking place alongside data collection.

## 3 RESULTS

Two overarching themes emerged through the thematic analysis of participant interviews, online survey, and documentary analysis: (a) Challenges in the implementation of learning health system pathways, and (b) benefits to the CHC approach for both staff and patients. Quotes from participants are used to exemplify and clarify themes. Due to under‐recruitment to the evaluation online survey and to further ensure participant anonymity due to small staff teams working on some care pathways and in some CHC regions, no further information regarding participant characteristics will be given.

### 3.1 Theme 1: Challenges in the implementation of learning health system pathways

#### 3.1.1 Subtheme: Time constraints

All participants noted the time constraints of trying to achieve an ambitious and innovative program of work within 2 years. This meant that for some they were still very much in the early stages of implementation and needed at least another year to fully implement and realize changes to care pathways at the time of this evaluation taking place. Delays in the signing off of funding contracts, allocation of funds, and putting staff in place meant that activities began to really get underway in January 2016, which gave the CHC programme considerably less time to implement the full program of work.

The majority of participants stated that the program as a whole had underestimated the scale of the challenge in getting NHS Trusts and HEIs with very different set‐ups, approaches, resources, and starting points to all get the degree of harmonization and staffing necessary to implement all that they wanted to do and this had led to delays in delivering outputs. Several participants commented that it took time to build trust and good working relationships between the different stakeholders, particularly in signing off governance arrangements for each care pathway:

*It's taken us 12 months to get all the ethics and agreements signed off. It just highlights how much longer and how difficult it is to get people to sign data sharing agreements when you don't have those relationships. We've had to build these from scratch as we've never worked with each before*.

Gaining the necessary approvals for the sharing of data had impacted all regions and caused significant delays. The time taken for sign off on data sharing agreements and applications to NHS Digital, NHS Trusts, and HEI research ethics approval ranged from 3 months for one pathway to 2 years in another. Participants noted that working directly with the NHS Trusts was key to gaining access to data quickly rather than requesting for data from NHS Digital. As one participant reflected on their experience:

*The willingness of the NHS Trusts, or most of the Trusts, to provide you with the data was amazing…compared to [name of pathway] where you're trying to get data from NHS Digital which is…er…just a pain in the arse really*.

#### 3.1.2 Subtheme: Data

All participants reported challenges in getting access to and receiving data, as well as data quality. Participants in three CHC regions reported that the data required for their pathway were not available in an electronic format, being stored in paper format in filing cabinets and in some cases, the paper files consisted of carbon copies of the original files. Elsewhere, participants from two pathways highlighted that data they had paid for had not yet been delivered resulting in one pathway reconfiguring their work to meet the new data specifications. Where participants had been able to gain deliveries of data, these were always late and not all data requests were delivered. Several participants reported that some NHS Trusts were reluctant to save data into the designated CHC Ark or Trusted Research Environment (TRE), despite all the necessary approvals having been granted, as there were still concerns about data security, despite CHC Ark systems exceeding security features of NHS organizations.

*I am amazed at how difficult it's been. It's been endless frustrations…you know we get approval at the senior level, then it goes down to middle management level and then it gets stopped and every time we get a change it gets stuck, so it's this endless cycle, like Dante's cycles of hell as we try to cope with these barriers that people have put in*.

Participants working with data expressed concerns about the data quality, highlighting the following specific issues: missing data, incorrectly coded data, and duplicated data. Criticisms were expressed that the data were paid for and in some cases, the data itself came from NHS Digital, and therefore, paid for data should be of better quality and standardized.

#### 3.1.3 Subtheme: Long‐term sustainability and commitment

There did not appear to be a consistent level of commitment from both DHSC as a funder and some partners within the CHC programme. The main challenge for each CHC region was to ensure that the partnership involved the right senior people in order to ensure commitment and direction at a high level. Each of the regions utilized a different governance structure, with mixed results. Only one region was successful from the start in fully implementing its governance structure, with senior staff in clearly defined roles, a clear regional vision, and operational staff, including dedicated project managers for each care pathway, had resulted in quicker progress made. Key issues that affected the other regions were a lack of clear vision and agreed set of regional objectives. Some staff noted that trying to get everyone to work out what was the common ground and then develop and implement strategies that would work for all was very time‐consuming.

All participants were concerned that all work completed to date will have been for short‐term gain rather than a long‐term investment in the North of England. Participants interviewed that did not work on the CHC programme noted the lack of secure data repositories for research purposes in the North of England besides the CHC programme and that DHSC needs to invest more in the North of England to reduce health inequalities:

*There are the issues about the sustainability of the CHC programme, especially if the Department of Health doesn't give us more funding…you know we're using it [data] now and there are costs associated with it…and there is a lot of nervousness of using it [data], especially after the end of the CHC programme*.

The short‐term nature of the CHC programme and lack of further investment from DHSC also affected staffing in all regions. Short‐term contracts and not being able to offer competitive salaries in line with industry and other sectors resulted in staffing shortages. As of December 2018, over half of staff employed on the CHC programme have left their posts due to the end of funding. This is resulting in some work being prioritized on what can be achieved with a skeletal team of staff.

*The time limited contracts have meant a lot of uncertainty so people are starting to move…so it's difficult to know who is going to be doing what types of work. I think when people have uncertainty about their own job prospects and roles, I think you need to devote a certain amount of your time to looking elsewhere and these projects, especially towards the tail end of these funded projects…it's quite difficult to focus on your work*.

#### 3.1.4 Subtheme: Different working cultures and priorities

Conflicts in the way different partners work were noted in all regions. In particular, the different pace of work and changing funding landscape between academia, the NHS, and industry created tensions within regions. Some of this was often due to the differences in language used by each partner, as one member of staff commented:

*We talk in different languages sometimes…but I think we've worked through that really…I remember we had a big discussion once about what a gate keeper was…and you think you're a [type of researcher] so you know what a gate keeper is, but it took us a long time to work out what a gate keeper is to other people…it's not impossible to understand it if you put it in the right language*.

Participants raised concerns that academia cannot keep up with the fast pace of delivery required by industry and other organizations (ie, CCGs, NHS trusts). However, industry and NHS organizations have appreciated the difficulties of the HEI working environment in terms of having to have information governance and HEI ethical approvals in place prior to receiving data into Arks and TREs.

#### 3.1.5 Subtheme: Communication

Internal communication was a challenge across the CHC programme. Getting all regions to communicate and cascade information had been especially difficult to achieve in some regions or within individual partner organizations. Many participants felt “disconnected” to the wider CHC programme, with communication blockages appearing both within regions, as well as overall as a program. As several participants commented, *“we don't seem to have the connected element of the 'Connected Health Cities' project”*. As a result, many participants felt isolated from both the regional and overall CHC programmes of work.

### 3.2 Theme 2: Benefits to the CHC approach for both staff and patients

#### 3.2.1 Subtheme: Benefits for staff involved in CHC programme

The CHC programme has provided HEIs, industry, and NHS organizations with the opportunity to collaborate. Participants noted that CHC has created opportunities to develop a new and innovative program of work that puts the needs of the patients and clinicians centrally. Being part of the CHC programme has resulted in a greater influence on tackling health inequalities within local populations in the North of England through the design, implementation, and governance arrangements.

The multidisciplinary approach utilized in all pathways and regions has resulted in a greater exposure to new research, theoretical concepts, and ways of working. Nearly all participants have taken advantage of this opportunity, resulting in a greater awareness of the problems faced in clinical practice and the potential use of health informatics in redesigning patient pathways:

*It's opened my eyes a little bit to the different methods, analytical methods and technologies in terms of how you would analyse large routine data sets and what you can do with them…I'm learning about what's possible making connections with people whom I would not have had interactions with*.

Furthermore, CHC funding has allowed partnerships to explore innovative ways of working with data without the constraints associated with traditional funding streams. As one participant reflected:

*It has allowed us to explore the possibilities more freely than what we would have been able to do. I think that having the funding there to do this has been fantastic, because it would be hard to find someone to fund something like this…you know, I don't think NIHR would have funded it*.

#### 3.2.2 Subtheme: Benefits for patients of CHC programme activities

Across all CHC regions, as well as the CHC programme as a whole, patients and members of the public have had an increased level of involvement. Participants working on care pathways noted how useful and beneficial it was to have insights from patients, members of the public, and health and social care staff in their projects. In some cases, the patient voice was key to pushing forward a piece of work when an NHS Trust might have been hesitant.

In other pathways, gathering patient and public views and engagement were seen as being critical elements of the CHC programme, to the extent that some regions have a specialist PPI role within their partnerships to enable the voice of patients to be heard. As one participant observed:

*I think that there should be an emphasis on patient and public involvement, especially when you're talking about data and information that belongs to patients…people talk about data within organisations, but that data belongs to patients and if you don't engage properly with them then you end up in trouble…and I think NHS England have been there before with a top‐down [approach], but I feel that CHC are doing this much better*.

At this point in the evaluation, it is too early to identify impact on patient outcomes from all CHC programme activities due to project delays. However, participants were able to reflect that the partnership approach has facilitated the faster roll‐out of research into clinical pathways, with pathway staff noting the accelerated rate of conducting research, testing outputs, and running small pilot studies compared to non‐CHC funded programs:

*[when you think about] how long it takes to roll it out, and it's something like 17 years from starting a project to rollout…compared to 18 months on this [CHC] pathway*.

## 4 DISCUSSION

The focus on developing learning health system in recent years has the potential to deliver better outcomes for patients and communities by analyzing routinely captured health information and feeding back results to clinical staff.^15^, ^16^ For a health system to be able to learn from the data it collects there is a need for a suitable infrastructure and working culture that supports the routine application of learning cycles.^17^ This suggests that both need to exist if a LHS is to be successful in practice.

This interim evaluation provides an in‐depth look at the key challenges of implementing a LHS at scale in the North of England. In particular, the CHC programme had to develop and implement both a LHS infrastructure and working culture across a range of organizations including academic institutions, NHS Trusts, industry partners, and data providers (such as NHS Digital) across four regions. Two overarching themes emerged through the thematic analysis of participant interviews, project documents and online survey: (a) challenges in the implementation of learning health system pathways, and (b) benefits to the CHC approach for both staff and patients.

In attempting to implement the CHC programme, the evaluation noted a number of challenges in implementing the CHC programme, which have been described in the results as “sub‐themes”. These include: t*ime constraints; data; long‐term sustainability and commitment; different working cultures and priorities; and communication*. While other LHS studies have described challenges in obtaining data from an accessibility perspective,^18^ here we have been able to provide further insight into the organizations in England that would need to be approached to gain approvals for data sharing in a LHS.

Challenges around data accessibility and quality in the CHC programme have also been discussed elsewhere. For example, issues around the heterogeneity of patient records, and differences between routine data and data collected for the purposes of research, means that data analysts cannot assume that patient data provides the full or accurate clinical picture of a care pathway, nor the population as a whole.^19^, ^20^ As noted in Reference 18, in their systematic review of adopting a LHS in practice, the CHC programme has found similar challenges in legal bases for data sharing agreements among participating organizations, building trust and follow‐on funding.

Despite the challenges that have been reported here, and in other LHS studies, we have been able to gain some early insight into the benefits of using a LHS. While it is too early in the delivery of the CHC programme to ascertain patient outcome impact, the multidisciplinary and collaborative approaches being used in CHC care pathways and regions have resulted in a greater exposure to new research, concepts, and ways of working with a deeper and nuanced understanding of the health of both regional and local populations. In addition, participants noted that gathering patient and public views and engagement were seen as being critical elements of enabling data sharing.

Our results presented here are based on data collected between May and October 2018 for what was originally deemed an end of program evaluation. However, halfway through the data collection period, DHSC changed the CHC programme deliverables and scope of evaluation. To enable this, the project as a whole was extended beyond the original end date of December 2018 to end of 2019. Furthermore, the majority of care pathways were still in their infancy due to ongoing delays in obtaining data approvals and there had been no baseline data collected at the start of the CHC programme. As a result, this evaluation changed from being an “end of program” evaluation to an interim evaluation. Therefore, a full end‐of‐program evaluation of the CHC programme would be able to consider the benefits, challenges and patient outcomes in greater depth.

On a more practical level, one of the key learnings from this interim evaluation has been the importance of building trust with all organizations involved in both care pathway and CHC region levels. Furthermore, participants noted the importance of working with and involving patients, members of the public and engaging with local populations as a whole in the use of patient data for health researcher. Participants stressed that in many cases, patient groups have helped drive the CHC programme of work forward and have provided vital input into study design. Time invested in building trust with both organizations and the public had contributed to the delay of the implementation of the infrastructure and working culture of CHC programme. In addition, the infrastructure, protocols, and governance arrangements that have been created by the CHC programme to date provide the foundation for future LHS projects and programs of work in the United Kingdom.

## 5 CONCLUSION

This article provides summary of the key challenges in implementing a LHS at scale in the North of England This interim evaluation found that while the CHC programme has encountered a range of challenges in implementing a LHS at scale, the program of work has contributed to the building of I.T and health informatics infrastructure in NHS organisations across the North of England. This could be exploited by future projects to further develop and expand each regional LHS to be more effective and efficient to further tackle health inequalities and improve health and wellbeing in the North of England and beyond.

## CONFLICT OF INTEREST

The authors declare no conflicts of interest.
